# Meta-Analysis of Wild Relatives and Domesticated Species of Rice, Tomato, and Soybean Using Publicly Available Transcriptome Data

**DOI:** 10.3390/life15071088

**Published:** 2025-07-11

**Authors:** Makoto Yumiya, Hidemasa Bono

**Affiliations:** 1 Graduate School of Integrated Sciences for Life, Hiroshima University, 3-10-23 Kagamiyama, Higashi-Hiroshima 739-0046, Japan; 2 Genome Editing Innovation Center, Hiroshima University, 3-10-23 Kagamiyama, Higashi-Hiroshima 739-0046, Japan

**Keywords:** domestication, meta-analysis, RNA-seq, wild relatives, domesticated species, rice, tomato, soybean

## Abstract

The domesticated species currently available in the market have been developed through the breeding of wild relatives. Breeding strategies using wild relatives with high genetic diversity are attracting attention as an important approach for addressing climate change and ensuring sustainable food supply. However, studies examining gene expression variation in multiple wild and domesticated species are limited. Therefore, we aimed to investigate the changes in gene expression associated with domestication. We performed a meta-analysis of public gene expression data of domesticated species of rice, tomato, and soybean and their presumed ancestral species using 21 pairs for rice, 36 pairs for tomato, and 56 pairs for soybean. In wild relatives, the expression of genes involved in osmotic, drought, and wound stress tolerance was upregulated, with 18 genes included in the top 5% of DW scores. In domesticated species, upregulated expression was observed in genes related to auxin and those involved in the efflux of heavy metals and harmful substances, with 36 genes included in the top 5% of DW scores. These findings provide insights into how domestication influences changes in crop traits. Thus, our findings may contribute to rapid breeding and the development of new varieties capable of growing in harsh natural environments. Hence, a new cultivation method called “de novo domestication” has been proposed, which combines the genetic diversity of currently unused wild relatives and wild relatives with genome editing technologies that enable rapid breeding.

## 1. Introduction

Owing to global warming, extreme weather events, and changes in the Earth’s environment, the yields of major global crops such as maize, rice, and soybeans could decrease 12–20% by the end of this century [1,2,3,4]. Additionally, with an increase in global population, food demand is expected to rise significantly, and by 2050, global food demand is projected to be approximately 1.5 times higher than that in 2010 [5]. To address these issues, the development of crop varieties with desirable traits in a short period is highly anticipated. In recent years, genome editing technologies, particularly CRISPR/Cas9, have garnered significant attention. Genome editing is a technology that enables the targeting of specific genes or nucleotide sequences to induce loss-of-function or introduce genes derived from other organisms. Using this genome editing technology, new varieties of various organisms, such as tomatoes with high gamma-aminobutyric acid content and red sea bream (Madai) with increased edible parts, have been developed [6,7]. Additionally, “de novo domestication” efforts utilizing wild relatives, which are genetic resources that have been underutilized until now, are also gaining attention for achieving sustainable crop production. Research cases of “de novo domestication” of wild tomatoes and wild rice have been reported in the literature [8,9,10]. The reason for this interest is because the currently distributed domesticated species have been selectively bred with a particular focus on yield, resulting in concerns about their low genetic diversity [11,12,13,14,15]. To implement such methods effectively, obtaining insights into the differences in traits between domesticated species and their wild counterparts is essential. However, studies examining gene expression variation in multiple wild and domesticated species are limited.

Meta-analysis is a valuable method that integrates multiple research findings to provide new insights. Meta-analyses utilizing public gene expression data have been performed and reported in the literature. For example, reports include a meta-analysis of soybean transcriptome data under heat, water, and drought stress [16], as well as an application that enables the identification and visualization of stress-responsive genes in *Arabidopsis thaliana* by applying analytical indices comparable to those used in the present study [17]. Additionally, the number of gene expression datasets registered in public databases is expected to increase in the future [18]. Therefore, the reliability of the analysis results is expected to increase as the number of datasets available for the meta-analysis increases. Given this background, we performed a meta-analysis using gene expression data from wild and domesticated species of rice, tomato, and soybean registered by multiple research groups in public databases to identify the gene groups specifically expressed in wild relatives and crops.

This study aimed to investigate the changes in gene expression associated with domestication and provide insights for developing new crop improvement strategies. Although the species analyzed in this study were not closely related and the number of gene expression datasets used was limited, this analysis provides valuable insights by focusing on changes in gene expression between wild and domesticated species during domestication.

## 2. Materials and Methods

### 2.1. Curation of Public Gene Expression Data

RNA sequencing (RNA-seq) data were obtained from public databases, primarily the National Center for Biotechnology Information Gene Expression Omnibus (NCBI GEO) [19]. To supplement the datasets not available in NCBI GEO, additional RNA-seq data were collected from published studies available online. In NCBI GEO, we performed searches using the scientific names of wild relatives, specifically “*Oryza rufipogon*” and “*Glycine soja*”. For tomato, two wild relatives, “*Solanum pennellii*” and “*Solanum arcanum*”, were utilized. The domesticated species paired with wild relatives and used in this analysis were “*Oryza sativa japonica*” for rice, “*Solanum lycopersicum*” for tomato, and “*Glycine max*” for soybean. To further refine the search results, a filter for “expression profiling by high-throughput sequencing” was applied. RNA-seq data were searched using the methods described above, and datasets containing paired wild and domesticated species from the same project were curated and used for subsequent analyses. The rationale for using data from the same project was to standardize the cultivation environments and experimental conditions as much as possible, thereby reducing batch effects.

### 2.2. Gene Expression Quantification

Each RNA-seq dataset was obtained using the prefetch (version 3.0.10) and Fasterq-dump (version 3.0.10) commands from the SRA Toolkit [20]. Quality control of the raw reads and removal of adapter sequences were performed using fastp (version 0.23.4) [21]. Subsequently, the output files from the quality check were aggregated into a single file using MultiQC (version 1.18) [22]. Transcripts were quantified using Salmon (version 1.10.1) [23]. For transcript quantification, reference cDNAs obtained from Ensembl Plant were used: *Oryza sativa japonica* and *Oryza rufipogon* with IRGSP-1.0; *Solanum pennellii*, *Solanum arcanum*, and *Solanum lycopersicum* with SL3.0; and *Glycine soja* and *Glycine max* with *Glycine_max_*v2.1. As a result, the quantified RNA-seq data were expressed as transcripts per million (TPM). Subsequently, transcript-level TPM values were summarized at the gene level using the tximport (version 1.28.0) [24] package in R.

### 2.3. Calculation of the DW Ratio

Gene expression data were normalized to the DW ratio. ‘D’ and ‘W’ represent ‘domesticated’ and ‘wild’, respectively. The DW ratio was calculated using the following equation:

DW Ratio = Domesticated TPM + 1/Wild TPM + 1(1)

When calculating the DW ratio, to avoid division by zero and prevent errors caused by genes with zero expression, 1 was added to the TPM values of both domesticated species and wild relatives.

### 2.4. Classification of Differentially Expressed Genes (DEGs) Based on the DW Ratio

To evaluate genes showing expression changes between domesticated species and their wild relatives, all genes were classified into three groups. Specifically, the three groups were upregulated, unchanged, and downregulated. These groupings were determined according to preestablished thresholds. Genes were classified as upregulated if their DW ratio exceeded an upper threshold, downregulated if their DW ratio fell below a lower threshold, and unchanged if they did not meet either of these criteria. For the upregulated category, 20 thresholds ranging from 1.5-fold to 200-fold were tested, and a 2-fold threshold was adopted. For the downregulated category, 20 thresholds ranging from 1/1.5 to 1/200 were tested, and a threshold of 1/2 was adopted [25].

### 2.5. Calculation of the DW Score

To evaluate DEGs by integrating different experiments based on the DW ratio, a DW score was calculated. The DW score was calculated by subtracting the number of pairs classified as upregulated from the number of pairs classified as downregulated. A pair refers to a set comprising one wild relative and one domesticated species from the same project.

DW Score = Downregulated pair count − Upregulated pair count(2)

The DW ratio and DW score were calculated using code from a previous study [26].

### 2.6. Gene Set Enrichment Analysis

Gene set enrichment analysis was performed using ShinyGO 0.81 [27] on the top- and bottom-ranking genes based on their DW scores. For the rice analysis, “*Oryza sativa japonica* Group gene IRGSP-1.0” was selected as the species, and “Gene Ontology (GO) Biological Process” was chosen as the pathway database. Default settings were used for all the other parameters. No enriched terms were observed when using species-specific annotations for tomato and soybean. Therefore, the gene IDs for both species were converted to *Arabidopsis thaliana* gene IDs, and enrichment analysis was performed using “*Arabidopsis thaliana* genes (TAIR10)”.

### 2.7. Commonly Upregulated Genes in Wild and Domesticated Species

To perform cross-species analysis, gene IDs from each species were converted to their corresponding *Arabidopsis thaliana* gene IDs (TAIR10). For this process, we used Ensembl Plant BioMart [28] to create a correspondence table linking the gene IDs of rice, tomato, and soybean with the gene IDs of *Arabidopsis thaliana* (TAIR10). Using the DW score for each species, we performed comparisons focusing on three ranges: the top 1%, 3%, and 5% of the upregulated and downregulated genes.

## 3. Results

### 3.1. Overview of the Study

**Figure 1:**
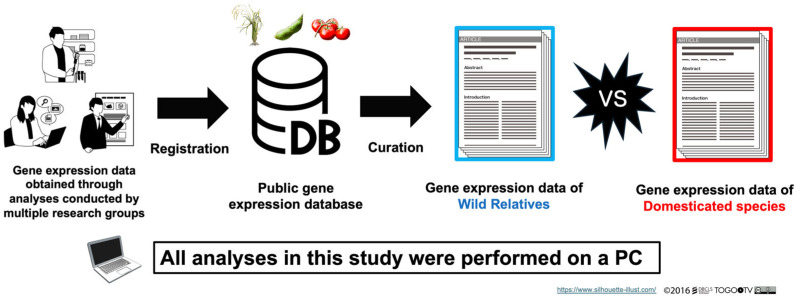
Gene expression data of wild relatives and domesticated species of rice, tomatoes, and soybeans were collected from public databases and manually curated. To ensure comparability, data only from wild and domesticated species included in the same research project were selected and paired. Subsequently, gene expression levels were quantified and differentially expressed genes between wild relatives and domesticated species were identified.

In this study, a meta-analysis was performed to identify DEGs using domesticated species and their ancestral wild relatives. Figure 1 presents an overview of the study.

### 3.2. Curation of RNA-Seq Data from Public Databases and the Literature

**Figure 2:**
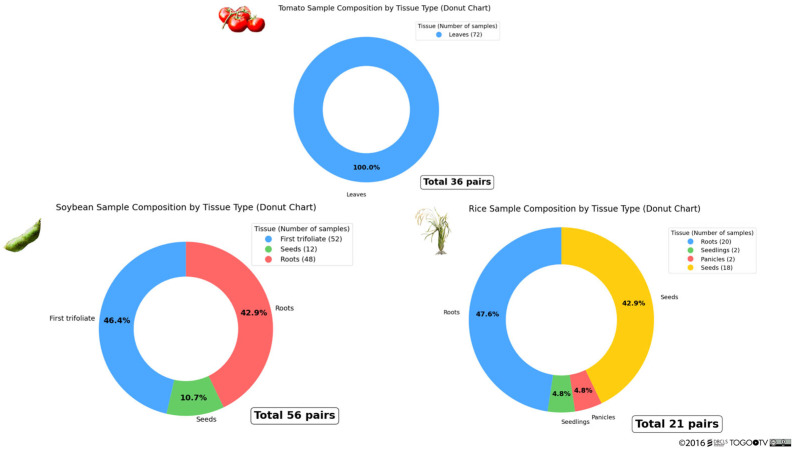
Number of plant samples and their tissue types used in this analysis. All tomato plant tissue samples were leaves, totaling 36 pairs. For soybean, the tissues included first trifoliates, seeds, and roots, with a total of 56 pairs. For rice, the tissues consisted of roots, seedlings, panicles, and seeds, totaling 21 pairs. Each pair comprised one sample from a wild relative and one sample from a domesticated species. Each sample was paired such that the wild and domesticated species shared the same tissue type and experimental conditions.

The RNA-seq data of wild and domesticated species used in this study were primarily collected from NCBI GEO [19]. Compared to domesticated species, data on wild relatives were substantially limited. Therefore, we decided to use three species—rice, tomato, and soybean—for the analysis because data from multiple projects were available for these species. The number of samples and their tissue types used in this analysis are shown in Figure 2.

To collect RNA-seq data, keyword searches were performed using the names of three wild relatives: *Oryza rufipogon*, *Solanum pennellii*/*Solanum arcanum*, and *Glycine soja*. RNA-seq data were obtained from five BioProjects in the NCBI GEO and used for analysis. However, because the number of available datasets from NCBI GEO was very limited, RNA-seq data were also collected from ArrayExpress in the European Bioinformatics Institute BioStudies [29] and the relevant literature. Ultimately, RNA-seq data from four BioProjects not registered in the NCBI GEO were obtained from the Sequence Read Archive [30], resulting in a total of nine BioProjects used for analysis. The metadata for the samples used in this analysis are provided in Supplementary Tables S1–S3, which correspond to *Oryza rufipogon* and *Oryza sativa japonica* (Table S1); *Solanum pennellii*, *Solanum arcanum*, and *Solanum lycopersicum* (Table S2); and *Glycine soja* and *Glycine max* (Table S3), respectively.

### 3.3. Classification of DEGs and Enrichment Analysis in Oryza rufipogon and Oryza sativa japonica

The expression quantification data for each species are provided in Supplementary Tables S4–S6, which correspond to *Oryza rufipogon* and *Oryza sativa japonica* (Table S4); *Solanum pennellii*, *Solanum arcanum*, and *Solanum lycopersicum* (Table S5); and *Glycine soja* and *Glycine max* (Table S6), respectively. Subsequently, both the expression ratio (DW ratio) and DW score of the wild and domesticated species were calculated. Based on the DW score ranking, lists of DEGs were obtained for both the wild and domesticated species. A threshold of 2-fold and 1.5-fold change was selected as the criterion for identifying genes with differential expressions. Lists of genes with DW scores above the 2-fold and 1.5-fold thresholds used in this analysis are provided in Supplementary Tables S7–S9, which correspond to *Oryza rufipogon* and *Oryza sativa japonica* (Table S7); *Solanum pennellii*, *Solanum arcanum*, and *Solanum lycopersicum* (Table S8); and *Glycine soja* and *Glycine max* (Table S9), respectively.

**Figure 3:**
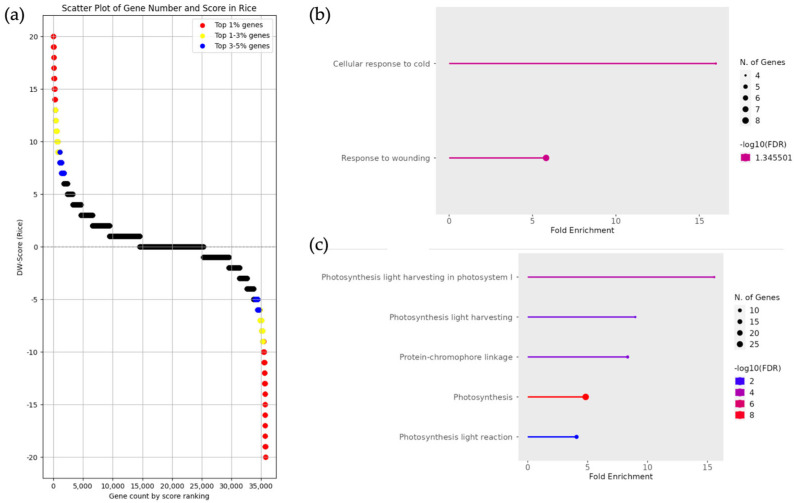
Enrichment analysis and DW score scatter plot of gene groups with upregulated expression in *Oryza sativa japonica* and *Oryza rufipogon*. (**a**) The scatter plot represents the DW score of each gene, where positive and negative scores indicate upregulated expression in *Oryza sativa japonica* and *Oryza rufipogon*, respectively. The red, yellow, and blue dots represent the top 1%, 1–3%, and 3–5% of scores, respectively. (**b**) Enrichment analysis results of gene groups with upregulated expression in *Oryza rufipogon*. The analysis was based on DW score, utilizing approximately the top 3% of genes, which included 898 genes (−20 ≦ DW score ≦ −7). (**c**) Enrichment analysis results of gene groups with upregulated expression in *Oryza sativa japonica*. The analysis was based on DW score, utilizing approximately the top 3% of genes, which included 1073 genes (9 ≦ DW score ≦ 20).

To investigate functional biases, we performed enrichment analysis on DEGs from pairs of *Oryza rufipogon* and *Oryza sativa japonica*, utilizing approximately the top 3% ranked using DW scores (Figure 3a). As a supplement, for rice, an enrichment analysis was initially performed using the top approximately 1% of the DEGs based on DW scores; however, no enriched terms were identified. Therefore, for the rice analysis only, the scope was expanded to include DEGs in approximately the top 3% based on DW scores. In the enrichment analysis targeting genes with upregulated expression in *Oryza rufipogon*, GO terms related to environmental responses, such as “cellular response to cold” and “response to wounding”, were found to be enriched (Figure 3b). In the results for *Oryza sativa japonica*, the GO terms related to photosynthesis and photosystems were enriched (Figure 3c). The genes included in the GO terms identified in *Oryza rufipogon* and *Oryza sativa japonica* are listed in Supplementary Tables S10 and S11, corresponding to *Oryza rufipogon* (Table S10) and *Oryza sativa japonica* (Table S11), respectively.

### 3.4. Classification of DEGs and Enrichment Analysis of Solanum pennellii, Solanum arcanum, and Solanum lycopersicum

In the enrichment analysis for tomato, as mentioned in the Section 2, tomato gene IDs were converted to *Arabidopsis thaliana* gene IDs, and the enrichment analysis was performed accordingly.

**Figure 4:**
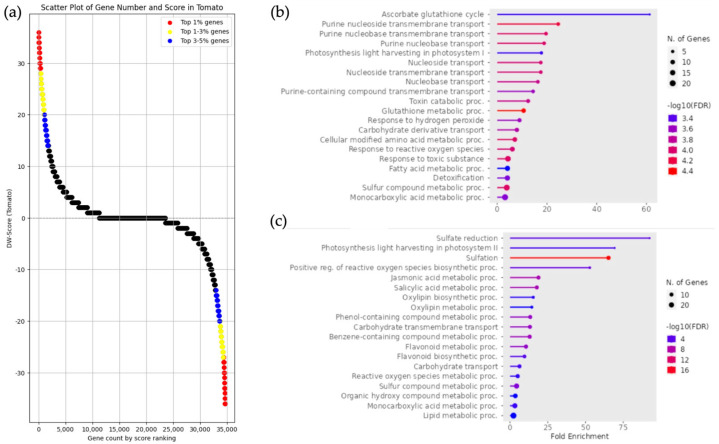
Enrichment analysis and DW score scatter plot of gene groups with upregulated expression in *Solanum lycopersicum*, *Solanum pennellii*, and *Solanum arcanum*. (**a**) The scatter plot represents the DW score of each gene, where positive and negative scores indicate upregulated expression in *Solanum lycopersicum* as well as *Solanum pennellii* and *Solanum arcanum*, respectively. The red, yellow, and blue dots represent the top 1%, 1–3%, and 3–5% of scores, respectively. (**b**) Enrichment analysis results of gene groups with upregulated expression in *Solanum pennellii* and *Solanum arcanum*. The analysis was based on DW score, utilizing approximately the top 1% of genes, which included 391 genes (−36 ≦ DW score ≦ −27). (**c**) Enrichment analysis results of gene groups with upregulated expression in *Solanum lycopersicum*. The analysis was based on DW scores, utilizing approximately the top 1% of genes, which included 382 genes (28 ≦ DW score ≦ 36).

Enrichment analysis was performed on DEGs from pairs of *Solanum pennellii*, *Solanum arcanum*, and *Solanum lycopersicum*, utilizing approximately the top 1% ranked using DW scores (Figure 4a). In the enrichment analysis of gene groups with upregulated expression in *Solanum pennellii* and *Solanum arcanum*, GO terms such as “ascorbate glutathione cycle” and “purine nucleoside transmembrane transport” were found to be enriched. Additionally, GO terms related to stress responses, such as “response to hydrogen peroxide” and “response to reactive oxygen species”, were also enriched (Figure 4b). In *Solanum lycopersicum*, GO terms related to sulfur metabolism and plant hormones, such as “sulfate reduction”, “jasmonic acid metabolic process”, and “salicylic acid metabolic process”, were enriched (Figure 4c). The genes included in the GO terms identified in *Solanum pennellii*, *Solanum arcanum*, and *Solanum lycopersicum* are listed in Supplementary Tables S12 and S13, corresponding to *Solanum pennellii* and *Solanum arcanum* (Table S12) and *Solanum lycopersicum* (Table S13), respectively.

### 3.5. Classification of DEGs and Enrichment Analysis in Glycine soja and Glycine max

Similar to tomato, soybean gene IDs were converted to *Arabidopsis thaliana* gene IDs, and enrichment analysis was performed.

**Figure 5:**
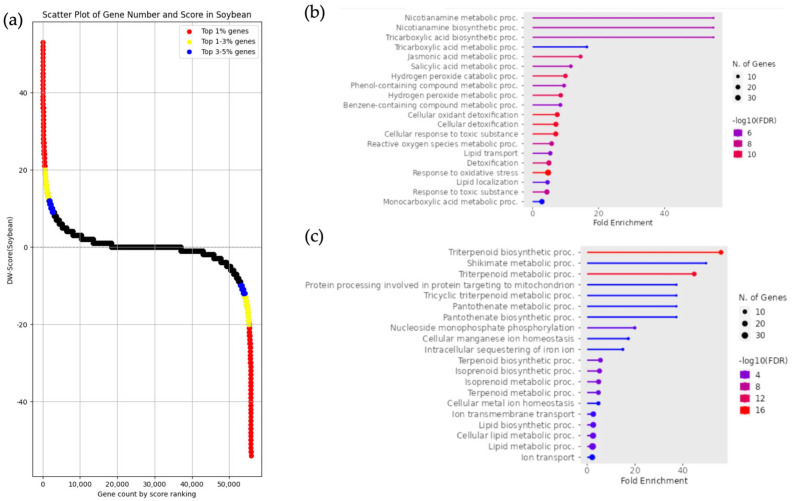
Enrichment analysis and DW score scatter plot of gene groups with upregulated expression in *Glycine max* and *Glycine soja*. (**a**) The scatter plot represents the DW score of each gene, where positive and negative scores indicate upregulated expression in *Glycine max* and *Glycine soja*, respectively. The red, yellow, and blue dots represent the top 1%, 1–3%, and 3–5% of scores, respectively. (**b**) Enrichment analysis results of gene groups with upregulated expression in *Glycine soja*. The analysis was based on DW score, utilizing approximately the top 1% of genes, which included 582 genes (−54 ≦ DW score ≦ −20). (**c**) Enrichment analysis results of gene groups with upregulated expression in *Glycine max*. The analysis was based on DW score, utilizing approximately the top 1% of genes, which included 590 genes (20 ≦ DW score ≦ 53).

Enrichment analysis was performed on DEGs from pairs of *Glycine soja* and *Glycine max* utilizing approximately the top 1% ranked using DW scores (Figure 5a). In *Glycine soja*, similar to the results observed in other wild relatives, GO terms such as “response to oxidative stress” and “cellular detoxification”, which contribute to defense mechanisms against environmental stress, were enriched. Additionally, a larger number of genes were classified under these terms (Figure 5b). In *Glycine max*, the enrichment analysis revealed that GO terms associated with secondary metabolites, such as triterpenoid and isoprenoid biosynthesis, as well as pathways related to energy metabolism, were enriched (Figure 5c). The genes included in the GO terms identified in *Glycine soja* and *Glycine max* are listed in Supplementary Tables S14 and S15, corresponding to *Glycine soja* (Table S14) and *Glycine max* (Table S15), respectively.

### 3.6. Common DEGs in Wild and Domesticated Species

To investigate genes commonly differentially expressed between wild and domesticated species of rice, tomato, and soybean, the gene IDs of each species were converted to *Arabidopsis thaliana* gene IDs. The correspondence tables for each gene ID created using BioMart [17] from Ensembl Plants focusing on the top 5% of genes by score for each species (Figures 3a–5a) are provided in Supplementary Tables S16–S21, corresponding to *Oryza rufipogon* (Table S16), *Oryza sativa japonica* (Table S17), *Solanum pennellii* and *Solanum arcanum* (Table S18), *Solanum lycopersicum* (Table S19), *Glycine soja* (Table S20), and *Glycine max* (Table S21), respectively.

**Figure 6:**
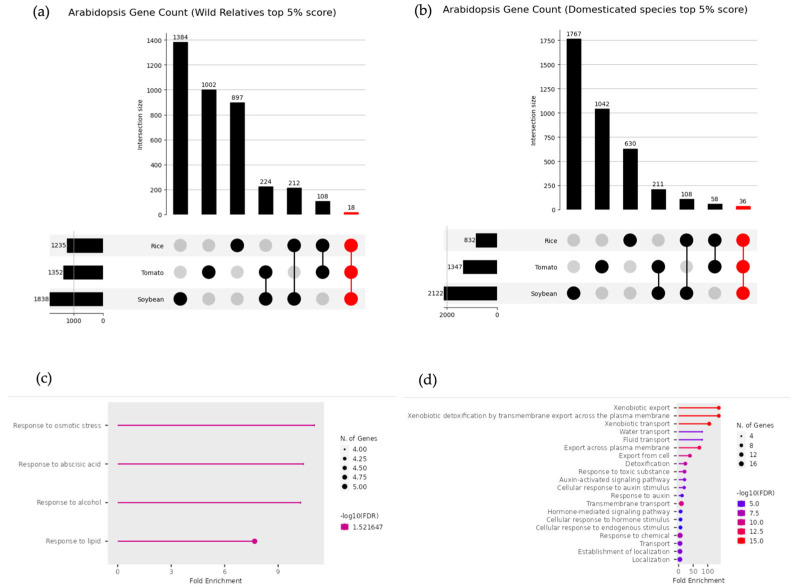
(**a**) UpSet plot of top 5% genes in wild relatives of rice, tomato, and soybean. (**b**) UpSet plot of top 5% genes in domesticated species of rice, tomato, and soybean. Enrichment analysis was performed on the top 5% of genes in both wild and domesticated species. (**c**) Enrichment analysis results for the 18 genes commonly upregulated in wild relatives. (**d**) Enrichment analysis results for the 36 genes commonly upregulated in domesticated species.

Eighteen genes were commonly upregulated in approximately the top 5% of DW scores for wild relatives, while 36 genes were identified for domesticated species (Figure 6a,b). These genes are listed in Table 1 and Table 2. Enrichment analysis results for the genes that were commonly upregulated in wild relatives and domesticated species are shown in Figure 6c,d. In wild relatives, GO terms related to environmental stress responses were enriched, consistent with the previous enrichment analysis results. However, in domesticated species, GO terms related to chemical compound export and detoxification, as well as the regulation of hormone responses, were enriched, differing from the enrichment analysis results observed in each individual domesticated species.

## 4. Discussion

In this study, we obtained gene expression data for wild and domesticated species of rice, tomato, and soybean from public databases and investigated the DEGs resulting from domestication effects. For this purpose, we utilized the DW ratio and DW score as analytical metrics to perform a meta-analysis comparing gene expression data from multiple research projects. Enrichment analysis was performed to investigate the characteristics of DEGs in both wild and domesticated species. Additionally, to identify genes that showed common differential expression across different species, the gene IDs of rice, tomato, and soybean were converted to *Arabidopsis thaliana* gene IDs. Based on these analyses, wild relatives included gene groups involved in environmental stress responses, enabling plants to adapt to harsh conditions. This finding supports the previously reported high environmental adaptability of wild relatives. In contrast, domesticated species contained genes involved in detoxification and export of chemical compounds. This is likely due, in large part, to the increased use of chemical fertilizers in crop cultivation. Based on these findings, the meta-analysis utilizing gene expression data from public databases suggests the high environmental adaptability of wild relatives and changes in crop traits and characteristics resulting from domestication.

### 4.1. Genes Commonly Upregulated Across Wild Relatives

In wild relatives, 18 genes were commonly upregulated within the top 5% of DW scores. The gene groups included in this analysis were associated with four GO terms: response to osmotic stress (GO:0006970), response to abscisic acid (GO:0001101), response to alcohol (GO:0097305), and response to lipid (GO:0033993), as shown in the enrichment analysis results in Figure 5c. A notable result is the enrichment of the abscisic acid response term, which plays a central role in responses to various stresses, such as drought, salinity, and low temperatures, across multiple species [31,32,33,34]. Additionally, GO terms related to the osmotic stress response and maintenance of cellular homeostasis were also enriched. Thus, the genes commonly upregulated in wild relatives included gene groups involved in environmental stress responses, suggesting that wild relatives possess a higher capacity to adapt to harsh natural environments than domesticated species.

Subsequently, we investigated whether the individual genes listed in Table 1—those commonly upregulated across wild relatives—have been previously reported to primarily function in environmental stress tolerance. *HKT1* is a key ion transporter involved in the plant salt stress response and salt tolerance. It primarily limits the translocation of Na^+^ from roots to leaves and stems, thereby contributing to the maintenance of ion homeostasis [35]. A study comparing salt tolerance between *HKT1* knockout plants and those with phloem-specific overexpression of *HKT1* reported that overexpression lines exhibited reduced Na^+^ translocation to the leaves, whereas knockout lines showed significant Na^+^ accumulation in the leaves. Additionally, plants with overexpressed *HKT1* produce more seeds and have higher overall yield under saline conditions compared to control plants [36]. Therefore, the molecular mechanism of salt tolerance mediated by *HKT1*, which exhibits upregulated expression in wild relatives, holds promise for developing crop varieties capable of thriving in saline-affected soils while maintaining superior yields.

The *RD22* gene functions as a molecular link connecting abscisic acid (ABA) signaling and abiotic stress responses and plays a critical role in plant drought stress adaptation [37]. In *Arabidopsis thaliana*, *RD22* exists as a single-copy gene, whereas certain plant species, such as grapevines, possess multiple paralogs, forming an expanded *RD22* family [38]. The expression of the *RD22* gene is regulated by two transcription factors. When plants are exposed to drought stress, ABA is synthesized in the initial phase, triggering the production of *MYB2* and *MYC2*—the transcription factors responsible for *RD22*. These factors promote *RD22* gene expression, resulting in the synthesis of *RD22* gene products that confer drought tolerance in plants [37,38,39,40,41].

The transcription factors *HB-7* and *HB-12* belong to the homeodomain-leucine zipper subfamily I. *HB-12* contributes to enhanced seed production under water stress conditions, while *HB-7* is involved in leaf development and photosynthesis promotion in mature plants [42]. These two genes are cooperatively regulated depending on developmental stages and environmental conditions, modulating processes associated with plant growth and water stress responses. *DOX1* is an enzyme that catalyzes the initial oxidation of fatty acids and possesses diverse functions, including pathogen defense, aphid-induced wound response, and protection against oxidative stress and cell death [43,44,45].

*MYB74* and *MYB102* belong to the R2R3-MYB transcription factor family and regulate stress responses and other plant-specific processes. *MYB102* is involved in the osmotic stress response and wound signaling pathway [46]. Specifically, in experiments investigating the feeding effects caused by *Pieris rapae* larvae, *MYB102* knockout mutants exhibited accelerated larval development rates and considerably higher pupation rates than control plants [47]. Thus, *MYB102* contributes to herbivory resistance. Similar to *MYB102*, *MYB74* is associated with environmental stress tolerance. Specifically, *MYB74* overexpression enhances osmotic stress tolerance. However, *MYB74* overexpression lines exhibit detrimental effects on growth compared with control plants [48]. These findings show that although *MYB74* overexpression enhances stress tolerance, particularly to osmotic stress, it negatively impacts plant growth. The present analytical results, showing higher *MYB74* expression in wild relatives and lower expression in domesticated species, suggest that domestication prioritizes yield. This implies a trade-off relationship; domesticated species lost the high stress tolerance inherent in wild relatives but gained increased yield, as supported by functional studies of *MYB74*.

In summary, the gene function analysis of wild relatives revealed that genes contributing to traits essential for survival in harsh environments, including biotic and environmental stresses, were highly expressed. Furthermore, the fact that genes highly expressed in wild relatives exhibited reduced expression in domesticated species suggests that domestication may have led to the gradual loss of these stress-resistance genes. Therefore, breeding approaches utilizing wild relatives—which retain the high stress tolerance lost in domesticated species—hold potential as valuable genetic resources for developing crop varieties adapted to increasingly harsh environmental conditions.

### 4.2. Genes Commonly Upregulated Across Domesticated Species

In domesticated species, the terms identified in the enrichment analyses of individual wild relatives were rarely observed. This difference can be attributed to the fact that rice, tomato, and soybean are not closely related species, and each species has undergone domestication and breeding to acquire different characteristics and traits. Conversely, the fact that genes involved in environmental stress tolerance were commonly detected even in analyses targeting non-closely related plant species suggests that these genes may play important roles across a wide range of plant species.

Next, we investigated the functions of genes that were commonly upregulated in domesticated species. The gene list for domesticated species included several auxin-related genes, such as *SHY2*, *IAA3*, *AXR3*, *IAA7*, *IAA14*, *IAA1*, and *ATAUX2-11*. Auxins are plant hormones involved in various functions, including the promotion of cell elongation and regulation of plant growth and development [49,50]. Although these genes have not been reported to directly contribute to increased crop yield, the observed upregulation of auxin-related genes in domesticated species probably reflects domestication-driven selection.

Among the genes showing upregulated expression in domesticated species, a particularly distinctive feature was the inclusion of multiple multidrug and toxic compound extrusion (MATE) family genes. *ALF5*, a member of the MATE family, confers resistance to tetramethylammonium. Furthermore, studies suggest that engineering plants to overexpress specific MATE proteins could enable their growth in chemically contaminated soils [51]. The MATE family genes that exhibited upregulated expression in this study were considerably enriched in GO terms related to chemical export and detoxification (Figure 6d), including detoxification (GO:0098754), response to toxic substance (GO:0009636), xenobiotic export (GO:0046618), xenobiotic detoxification by transmembrane export across the plasma membrane (GO:1990961), xenobiotic transport (GO:0042908), export from cell (GO:0140352), and export across plasma membrane (GO:0140115). One of the genes included in these terms, *DTX1*, mediates the efflux of the heavy metal cadmium as well as toxic compounds [52]. One possible reason for the increased expression of MATE gene family members in domesticated species is the change in the soil environment resulting from the increased use of chemical fertilizers. The adverse effects of heavy metal and pesticide accumulation in modern agricultural soils on plant growth and health have been extensively documented in scientific studies [53] Considering these factors, agricultural environments for domesticated species, domesticated from wild ancestors, exhibit elevated heavy metal accumulation in soils compared to traditional systems, driven by increased agrochemical use (e.g., pesticides and chemical fertilizers) and pollution linked to modern agricultural practices. Consequently, we hypothesized that domesticated species exhibit an upregulated expression of genes associated with the detoxification and export of harmful substances, a phenomenon that is not observed in their wild counterparts. However, most MATE family genes, other than *DTX1*, remain unnamed and are likely to be understudied, warranting further detailed functional analyses.

*XTH32*, another gene showing upregulated expression in domesticated species, is a member of the enzyme family involved in plant cell wall remodeling. It catalyzes the cleavage and polymerization of xyloglucan, thereby contributing to plant growth and development [54]. Therefore, *XTH32* probably contributes to the development of large-yielding individuals commonly observed in domesticated species. Furthermore, this gene family is involved in adaptation to external stresses [55] and is mentioned in meta-analyses of *Arabidopsis* abiotic stress responses performed using methods similar to this analysis [56,57].

This study has several limitations that need to be taken into consideration. First, the number of datasets used for the meta-analysis was small, which may have introduced bias in the results. This is due to the current scarcity of research projects in public databases that include RNA-seq data for both wild and domesticated species. This issue will likely be resolved in the future when studies comparing wild and domesticated species are conducted and their respective RNA-seq data accumulate. Second, well-annotated transcriptome data for wild relatives were lacking. The reference transcriptome used to quantify expression levels was derived from domesticated species. Consequently, the accuracy of gene expression quantification and identification of specific genes in wild relatives may have been compromised. To address this issue, the establishment of high-quality data for wild relatives is essential. Third, when comparing genes with variable expression across different species, the analysis was standardized to *Arabidopsis* gene names. However, because tomato, rice, and soybean are not closely related, several genes could not be matched. Consequently, genes exhibiting variations in expression in both wild and domesticated species may not be reflected in the results of this study. This issue is likely to occur when standardizing gene names to those of specific species. However, improvements are expected as the identification of gene functions advances in many species beyond model organisms. Finally, because the identification of genes with different expression levels in this study did not involve statistical testing, the results should be interpreted with caution. Despite these limitations, this study is a valuable resource for understanding trait changes due to domestication and for applications in breeding, given that few studies have examined gene expression variation in multiple wild and domesticated species.

## 5. Conclusions

A meta-analysis involving multiple wild relatives and domesticated species enabled the identification of DEGs associated with the effects of domestication. In wild relatives, increased expression of multiple genes that contribute to environmental stress tolerance was observed. These findings suggest that wild relatives have the potential to grow and survive in harsh environmental conditions. This finding also supports recent trends in breeding methods that utilize valuable traits of wild relatives. In contrast, in domesticated species, particularly rice, an upregulation of photosynthesis-related genes was observed. Since photosynthesis is closely associated with crop yield, this result may reflect the selection and breeding of high-yielding individuals during the domestication process. In the three domesticated species, the genes commonly upregulated included those involved in chemical response, efflux, and detoxification. These results reflect the impact of increased agrochemical use (e.g., pesticides and chemical fertilizers) on contemporary crop cultivation. Additionally, auxin-related genes and enzymes involved in cell-wall remodeling were identified among the upregulated genes in domesticated species, suggesting their potential contribution to yield improvement through domestication-driven selection.

The findings of this study provide valuable insights into the genetic basis of crop domestication and contribute to the identification of candidate genes for breeding that are expected to be applied in future crop improvement efforts. In particular, the utilization of wild relatives to develop new crop varieties holds significant potential as a valuable approach for addressing various challenges in future crop breeding. Although the number of species and samples used in this analysis was limited, combining analyses involving a greater diversity of species with emerging scientific technologies such as genome editing is expected to enable the development of crop varieties with desirable traits in a shorter timeframe than that required for traditional methods.

## Figures and Tables

**Table 1:** Genes commonly upregulated in wild relatives (*Oryza rufipogon*, *Solanum pennellii, Solanum arcanum*, and *Glycine soja*).

Gene Stable ID	Gene Name	Description
AT1G10400	AT1G10400	UDP-glycosyltransferase superfamily protein
AT1G10810	AT1G10810	NAD(P)-linked oxidoreductase superfamily protein
AT1G23800	ALDH2B7	aldehyde dehydrogenase 2B7
AT1G60680	AT1G60680	NAD(P)-linked oxidoreductase superfamily protein
AT1G60690	AT1G60690	NAD(P)-linked oxidoreductase superfamily protein
AT1G60710	ATB2	NAD(P)-linked oxidoreductase superfamily protein
AT1G60730	AT1G60730	NAD(P)-linked oxidoreductase superfamily protein
AT1G60750	AT1G60750	NAD(P)-linked oxidoreductase superfamily protein
AT2G16890	AT2G16890	UDP-glycosyltransferase superfamily protein
AT2G46370	JAR1	Auxin-responsive GH3 family protein
AT2G46680	HB-7	Homeobox 7
AT3G01420	DOX1	Peroxidase superfamily protein
AT3G61890	HB-12	Homeobox 12
AT4G05100	MYB74	MYB domain protein 74
AT4G10310	HKT1	High-affinity K + transporter 1
AT4G21440	MYB102	MYB-like 102
AT5G14860	AT5G14860	UDP-glycosyltransferase superfamily protein
AT5G25610	RD22	BURP domain-containing protein

**Table 2:** Genes commonly upregulated in domesticated species (*Oryza sativa japonica*, *Solanum lycopersicum*, and *Glycine max*).

Gene Stable ID	Gene Name	Description
AT1G04240	SHY2	AUX/IAA transcriptional regulator family protein
AT1G04250	AXR3	AUX/IAA transcriptional regulator family protein
AT1G10960	FD1	Ferredoxin 1
AT1G20930	CDKB2;2	Cyclin-dependent kinase B2;2
AT1G60950	FD2	2Fe-2S ferredoxin-like superfamily protein
AT1G64590	AT1G64590	NAD(P)-binding Rossmann-fold superfamily protein
AT1G64820	AT1G64820	MATE efflux family protein
AT1G66760	AT1G66760	MATE efflux family protein
AT1G66780	AT1G66780	MATE efflux family protein
AT1G76540	CDKB2;1	Cyclin-dependent kinase B2;1
AT2G04040	DTX1	MATE efflux family protein
AT2G04050	AT2G04050	MATE efflux family protein
AT2G04066	AT2G04066	MATE efflux family protein
AT2G04070	AT2G04070	MATE efflux family protein
AT2G04080	AT2G04080	MATE efflux family protein
AT2G04090	AT2G04090	MATE efflux family protein
AT2G04100	AT2G04100	MATE efflux family protein
AT2G13610	ABCG5	ABC-2 type transporter family protein
AT2G16850	PIP2;8	Plasma membrane intrinsic protein 2;8
AT2G36870	XTH32	Xyloglucan endotransglucosylase/hydrolase 32
AT2G42380	BZIP34	Basic-leucine zipper (bZIP) transcription factor family protein
AT2G48020	AT2G48020	Major facilitator superfamily protein
AT3G23030	IAA2	Indole-3-acetic acid inducible 2
AT3G23050	IAA7	Indole-3-acetic acid 7
AT3G58120	BZIP61	Basic-leucine zipper (bZIP) transcription factor family protein
AT4G14550	IAA14	Indole-3-acetic acid inducible 14
AT4G14560	IAA1	Indole-3-acetic acid inducible
AT4G17340	TIP2;2	Tonoplast intrinsic protein 2;2
AT4G25000	AMY1	Alpha-amylase-like protein
AT4G35100	PIP3	Plasma membrane intrinsic protein 3
AT5G10130	AT5G10130	Pollen Ole e 1 allergen and extension family protein
AT5G10180	SULTR2;1	Sulfate transporter 2;1
AT5G10570	AT5G10570	Basic helix-loop-helix (bHLH) DNA-binding superfamily protein
AT5G43700	ATAUX2-11	AUX/IAA transcriptional regulator family protein
AT5G47450	TIP2;3	Tonoplast intrinsic protein 2;3
AT5G65640	bHLH093	Beta HLH protein 93

## Data Availability

The original data presented in the study are openly available in FigShare at https://doi.org/10.6084/m9.figshare.c.7801100.v1.

## Supplementary Materials

The following supporting information can be downloaded at: https://doi.org/10.6084/m9.figshare.c.7801100.v1. Table S1: *Oryza rufipogon* and *Oryza sativa japonica* sample metadata. Table S2: *Solanum pennellii, Solanum arcanum,* and *Solanum lycopersicum* sample metadata. Table S3: *Glycine soja* and *Glycine max* metadata. Table S4: TPM Data of *Oryza rufipogon* and *Oryza sataiva japonica*. Table S5: TPM data of *Solanum pennellii*, *Solanum arcanum,* and *Solanum lycopersicum*. Table S6: TPM data of *Glycine soja* and *Glycine max*. Table S7: DW scores for *Oryza rufipogon* and *Oryza sataiva japonica*. Table S8: DW scores for *Solanum pennellii, Solanum arcanum,* and *Solanum lycopersicum*. Table S9: DW scores for *Glycine soja* and *Glycine max*. Table S10: Gene list for *Oryza sativa rufipogon* enrichment analysis. Table S11: Gene list for *Oryza sativa japonica* enrichment analysis. Table S12: Gene list for *Solanum pennellii* and *Solanum arcanum* enrichment analysis. Table S13: Gene list for *Solanum lycopersicum* enrichment analysis. Table S14: Gene list for *Glycine soja* enrichment analysis. Table S15: Gene list for *Glycine max* enrichment analysis. Table S16: *Arabidopsis thaliana*–*Oryza rufipogon* DW score Top5 Percent_Gene_Correspondence. Table S17: *Arabidopsis thaliana*–*Oryza sativa japonica* DW score Top5 Percent_Gene_Correspondence. Table S18: *Arabidopsis thaliana*–*Solanum pennellii* and *Solanum arcanum* DW score Top5 Percent_Gene_Correspondence. Table S19: *Arabidopsis thaliana*–*Solanum lycopersicum* DW score Top5 Percent_Gene_Correspondence. Table S20: *Arabidopsis thaliana*–*Glycine soja* DW score Top5 Percent_Gene_Correspondence. Table S21: *Arabidopsis thaliana*–*Glycine max* DW score Top5 Percent_Gene_Correspondence.

## Abbreviations

The following abbreviations are used in this manuscript:

RNA-seq	RNA sequencing
NCBI GEO	National Center for Biotechnology Information Gene Expression Omnibus
TPM	Transcripts per million
DW	Domesticated and wild
DEG	Differentially expressed gene
GO	Gene Ontology
ABA	Abscisic acid
MATE	Multidrug and toxic compound extrusion
